# Quantitative Analysis of Propranolol Hydrochloride by High Performance Thin Layer Chromatography

**DOI:** 10.4103/0250-474X.43016

**Published:** 2008

**Authors:** Girija Bhavar, V. A. Chatpalliwar

**Affiliations:** Department of Pharmaceutical Chemistry, R. C. Patel College of Pharmacy, Karvand Naka, Shirpur-425 405, India

**Keywords:** HPTLC, propranolol hydrochloride, tablets, validation

## Abstract

A simple, accurate and precise HPTLC method has been developed for estimation of propranolol hydrochloride from bulk drug and tablet formulations. The separation was achieved on TLC plates using appropriate solvent system. The spots so developed were densitometrically scanned at 290 nm. The linearity of the method was found to be within the concentration range of 200–2000 ng/spot. The validation parameters, tested in accordance with the requirements of ICH guidelines, prove the suitability of this method. The method was successively applied for determination of drug in tablets, wherein, no interference from tablet excipients was observed.

Propranolol hydrochloride, chemically 1-[(1-methylethyl)amino]-3-(1-naphthylenyloxy)-2-propranolol hydrochloride^1^, is a non-selective β-adrenergic antagonist. It is used in the management of hypertension, phaeochromocytoma, angina pectoris, myocardial infarction and cardiac arrhythmias^2^. Literature reported several titrimetric^3^, complexation^4^ and spectrometric^5^ methods for analyzing propranolol. Several HPLC methods have also been reported for determining propranolol in pharmaceutical formulations and in biological fluids 6–14. Indian Pharmacopoeia describes a spectrometric method^15^, whereas, USP describes a HPLC method^16^ for assaying the drug. Following description is of a rapid, reliable and accurate HPTLC method for determination of propranolol hydrochloride in tablets.

All chemicals used were of analytical reagent grade, purchased from M/s Qualigens Fine Chemicals Ltd., Mumbai, India. The tablets were procured from the local market. HPTLC system consisted of Camag Linomat V applicator (Muttenz, Switzerland), a Camag twin trough TLC chamber of appropriate size, Camag TLC scanner 3, Wincats software (version 1.3.0.) and a Hamilton syringe (Switzerland) of 100 μl capacity.

Chromatography was performed on 20×10 cm aluminium backed silica gel 60 F_254 TLC_ plates (Merck, Darmstadt, Germany). Samples, applied as solutions of appropriate concentrations using Hamilton micro syringe and Linomat V applicator (Camag, Muttenz, Switzerland) were 6 mm wide bands with a distance of 13 mm within each spot. The plate was developed by ascending movement of solvent system, for 70 mm from the point of application, in a Camag twin trough chamber which was previously saturated with vapors of solvent system for a pre-optimized time period of 20 min at 25°±2°. The solvent system consisted of isopropanol:ethyl acetate:ammonia (1:8.5:0.5 v/v/v). The plate was dried and scanned at 290 nm, using Camag TLC scanner 3, equipped with Wincats software version 1.3.0., and slit dimensions of the scanner being set at 6.00 × 0.45 mm.

A stock solution containing 200 μg/ml of propranolol hydrochloride was prepared in methanol. Aliquots of this solution were applied on the plates to give spots with concentrations of 200-2000 ng/spot. Each concentration was applied six times and developed as described above. Chromatograms were obtained representing sharp and symmetrical peak. Calibration curve was established by plotting the obtained peak area on ordinate against corresponding concentration on abscissa.

To determine the content of the drug in solid dosage form, 20 tablets were accurately weighed, their average weight calculated and they were finely powdered. Powder equivalent to 40 mg of the drug was dissolved in methanol and sonicated for 20 min. The solution was filtered and diluted to 100 ml with the same solvent. An aliquot measuring 1 ml was diluted to 10 ml with methanol and this solution was appropriately applied on the plate to get approximately 400 ng/spot of the drug. The results obtained are summarized in Table 1. The method was validated as per ICH guidelines^17^.

**TABLE 1:** ASSAY OF PROPRANOLOL HYDROCHLORIDE IN TABLET

Label claim	40 mg
Amount found ±SD (n=6)	40.27±0.254 mg
% Label claim	100.68 %
% RSD	0.630

n is number of repetitions

A standard stock solution of the drug was applied on the plate so as to contain 200-2000 ng of propranolol hydrochloride per spot. The plate was developed, dried, and scanned as described above. All measurements were repeated 6 times for each concentration. Correlation coefficient (r) of the line, passing through all the points on a graph constructed by plotting mean of peak areas on ordinate against corresponding concentrations on ordinate, was found to be 0.9997 with slope 4.905.

Limit of detection (LOD) and limit of quantitation (LOQ) were studied to determine the sensitivity of the developed method. LOD was calculated using formula, LOD= 3.3×σ/S, where, σ is residual standard deviation of regression line and S is slope of corresponding line. The LOD and LOQ were found to be 59.72 ng and 180.97 ng of the drug, respectively.

To ensure accuracy of the method, recovery studies were performed by standard addition method at 80%, 100% and 120% level, to the pre-analyzed samples and the subsequent solutions were re-analyzed. At each level, three determinations were performed and the results obtained are shown in Table 2. The results of recovery studies were within the specified limits of ICH guidelines. Lower values of % RSD reflect the accuracy of the method.

**TABLE 2:** RESULTS OF RECOVERY STUDIES

Drug added (ng)	% Recovery n=3	Amount recovered (ng)±SD	% RSD
320	100.61	321.97±1.155	0.359
400	99.97	399.87±1.938	0.485
480	99.96	479.83±2.040	0.425

n is number of repetitions

Precision, expressed in terms of % RSD, was determined in terms of intra-day and Inter-day precisions, analyzing the drug at three different concentrations, determining each concentration thrice. The sample solutions were analyzed using the method for 3 consecutive days, repeating the process twice-a-day at different period. The results obtained are summarized in Table 3, and reflect high degree of precision.

**TABLE 3:** PRECISION OF THE METHOD

Amount applied (ng)	Intra-day precision (% RSD) n=3	Inter-day precision (% RSD) n=3
400	0.535	0.459
800	0.145	0.148
1200	0.051	0.077

n is number of repetitions

Two different analyst performed assay on marketed tablets of the drug, in similar operational and environmental conditions, using the developed method to determine its ruggedness, and the results are summarized in Table 4. The optimized solvent system yielded a symmetrical peak for the drug with R_f_ 0.52 (fig. 1). A typical absorbance spectrum of the drug is shown in fig. 2. The peak for the drug from tablets was identified by comparing the R_f_, and also comparing its absorbance spectrum with that obtained with the standard drug. Peak purity was determined by co-relating the spectra of drug at peak start (s), peak apex (m) and at peak end (e) positions, (correlation r (s, m) = 0.9999, r (m, e) = 0.9997). Thus, it can be concluded that no impurities or degradation products, likely to be present in the marketed tablets, were found to interfere with the peak of the drug.

**TABLE 4:** RUGGEDNESS TEST FOR THE METHOD

Parameter	Analyst 1 (n=6)	Analyst 2 (n=6)
Amount applied (ng)	400	400
Amount found (ng)	401.57	401.46
% RSD	0.273	0.287

n is number of replicates

**Fig. 1:**
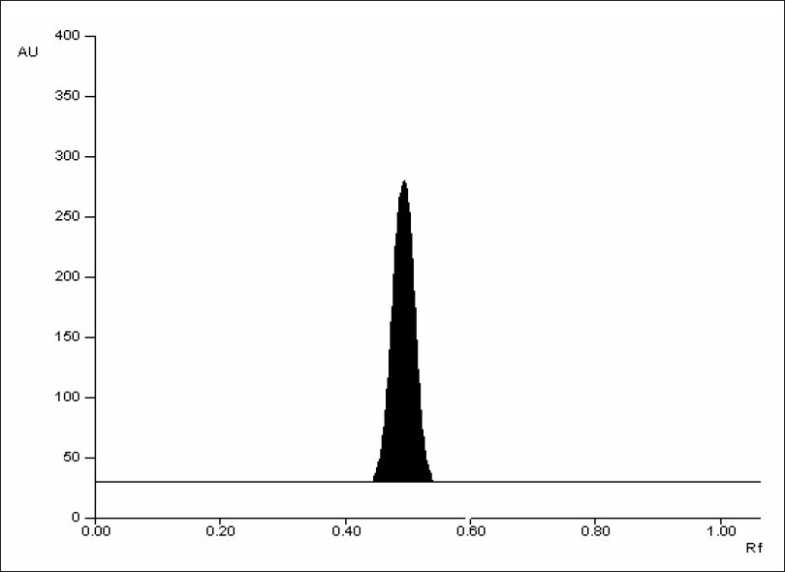
A typical chromatogram of propranolol hydrochloride scanned at 290 nm

**Fig. 2:**
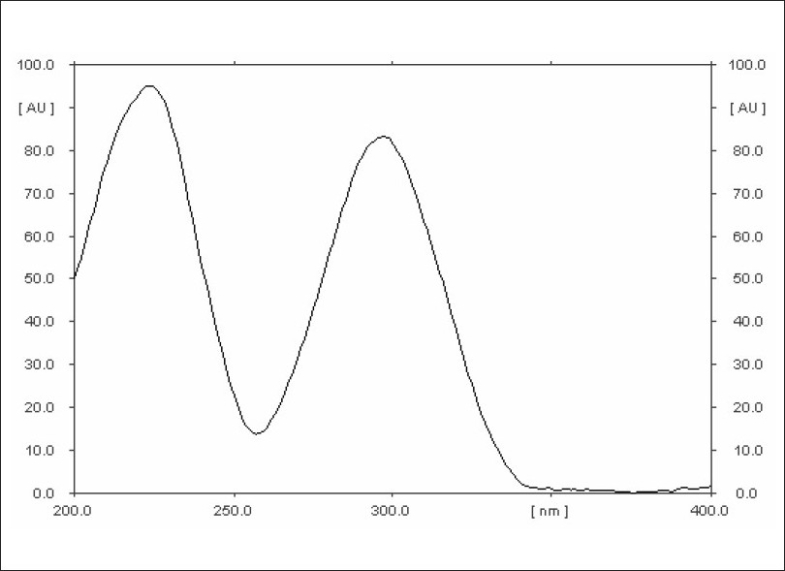
A typical absorption spectrum of propranolol hydrochloride scanned at 200-400 nm
